# Secondary Unconjugated Bile Acids Induce Hepatic Stellate Cell Activation

**DOI:** 10.3390/ijms19103043

**Published:** 2018-10-05

**Authors:** Kunihiro Saga, Yukio Iwashita, Shinya Hidano, Yuiko Aso, Kenji Isaka, Yasutoshi Kido, Kazuhiro Tada, Hiroomi Takayama, Takashi Masuda, Teijiro Hirashita, Yuichi Endo, Masayuki Ohta, Takashi Kobayashi, Masafumi Inomata

**Affiliations:** 1 Department of Gastroenterological and Pediatric Surgery, Faculty of Medicine, Oita University, 1-1 Idaigaoka, Hasama-machi, Oita 879-5593, Japan; iwashita@oita-u.ac.jp (Y.I.); asoyui.experi@gmail.com (Y.A.); m1341101@oita-u.ac.jp (K.I.); kaztada@oita-u.ac.jp (K.T.); t-1603@oita-u.ac.jp (H.T.); tmasuda@oita-u.ac.jp (T.M.); teij03@oita-u.ac.jp (T.H.); endo@oita-u.ac.jp (Y.E.); ohta@oita-u.ac.jp (M.O.); inomata@oita-u.ac.jp (M.I.); 2 Department of Infectious Disease Control, Faculty of Medicine, Oita University, 1-1 Idaigaoka, Hasama-machi, Oita 879-5593, Japan; hshinya@oita-u.ac.jp (S.H.); takashik@oita-u.ac.jp (T.K.); 3 Department of Environmental and Preventive Medicine, Faculty of Medicine, Oita University, 1-1 Idaigaoka, Hasama-machi, Oita 879-5593, Japan; kidoyasu@oita-u.ac.jp

**Keywords:** hepatic stellate cell, secondary unconjugated bile acid, DNA microarray, tumor necrosis factor signaling pathway

## Abstract

Hepatic stellate cells (HSCs) are key players in liver fibrosis, cellular senescence, and hepatic carcinogenesis. Bile acids (BAs) are involved in the activation of HSCs, but the detailed mechanism of this process remains unclear. We conducted a comprehensive DNA microarray study of the human HSC line LX-2 treated with deoxycholic acid (DCA), a secondary unconjugated BA. Additionally, LX-2 cells were exposed to nine BAs and studied using immunofluorescence staining, enzyme-linked immunosorbent assay, and flow cytometry to examine the mechanisms of HSC activation. We focused on the tumor necrosis factor (TNF) pathway and revealed upregulation of genes related to nuclear factor kappa B (NF-κB) signaling and senescence-associated secretory phenotype factors. α-Smooth muscle actin (α-SMA) was highly expressed in cells treated with secondary unconjugated BAs, including DCA, and a morphological change associated with radial extension of subendothelial protrusion was observed. Interleukin-6 level in culture supernatant was significantly higher in cells treated with secondary unconjugated BAs. Flow cytometry showed that the proportion of cells highly expressing α-SMA was significantly increased in HSCs cultured with secondary unconjugated BAs. We demonstrated that secondary unconjugated BAs induced the activation of human HSCs.

## 1. Introduction

Hepatic stellate cells (HSCs) are present in Disse’s cavities around the sinusoids. In normal liver, HSCs store vitamin A and are in a quiescent state. However, in response to liver injury induced by conditions such as obesity, viral infection, alcohol intoxication, or oxidative stress, HSCs transform into an activated state, characterized by changes in cell morphology, gene expression, and secretion of chemokines [1,2,3,4,5,6]. Activated HSCs cannot store retinoids, and they produce type I collagen, matrix metalloproteinases, and alpha-smooth muscle actin (α-SMA), promoting liver fibrosis due to inflammation [7].

It was recently reported that HSCs activated due to obesity cause cellular senescence and secrete inflammatory cytokines, thereby promoting carcinogenesis of the surrounding hepatocytes [8]. One of the mechanisms via which obesity predisposes to liver cancer is a change in the intestinal bacterial flora [9]. Increased levels of *Clostridium* cluster XI and XIVa and Gram-positive bacteria in the intestinal flora are frequently observed in obese individuals [8]. As the number of these bacteria increases, primary bile acids (BAs) are more likely to be decomposed into secondary BAs in the intestine, resulting in the increased proportion of secondary BAs entering the liver via enterohepatic circulation [8]. Additionally, lipoteichoic acid (LTA), a component of Gram-positive gut microbes, was reported to co-enhance the activation of HSCs with secondary BAs, thereby upregulating the expression of senescence-associated secretory phenotype (SASP) factors [10].

**Figure 1:**
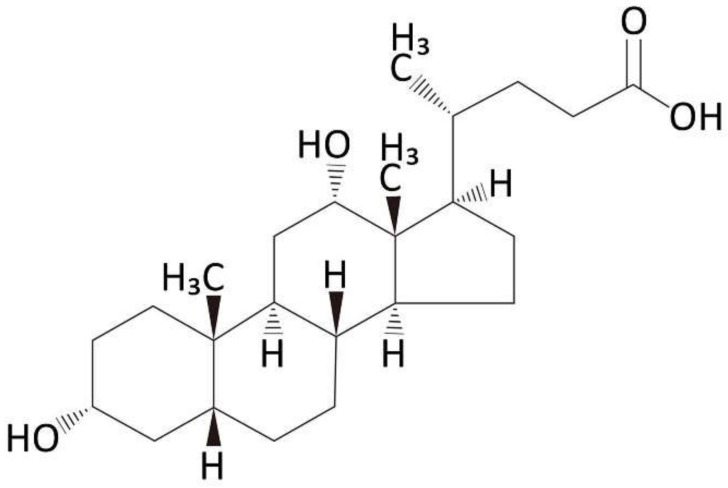
Chemical structure of deoxycholic acid (DCA). The molecular weight of DCA is 392.572 g/mol.

BAs are classified into primary conjugated BAs, primary unconjugated BAs, secondary conjugated BAs, and secondary unconjugated BAs. However, it is not yet clarified which BAs cause differentiation of HSCs and to what extent. Here, using DNA microarrays, we comprehensively analyzed gene expression changes in the human HSC line following their co-exposure to deoxycholic acid (DCA) (Figure 1), a secondary BA, and LTA. In addition, we investigated the morphological changes and degrees of activation and senescence of HSCs stimulated with various BAs (Table 1).

## 2. Results

### 2.1. DNA Microarray

Whole-genome microarray analysis of the expression profiles of 58,201 genes was carried out to identify gene expression levels in control LX-2 cells and those treated with DCA and LTA. This analysis revealed 5390 genes with statistically significant changes due to the treatment. Among these, 2457 genes with at least twofold higher expression levels were considered positively regulated by DCA and LTA, whereas 2933 genes in which expression levels were twofold lower were considered negatively regulated by the treatment.

**Figure 2:**
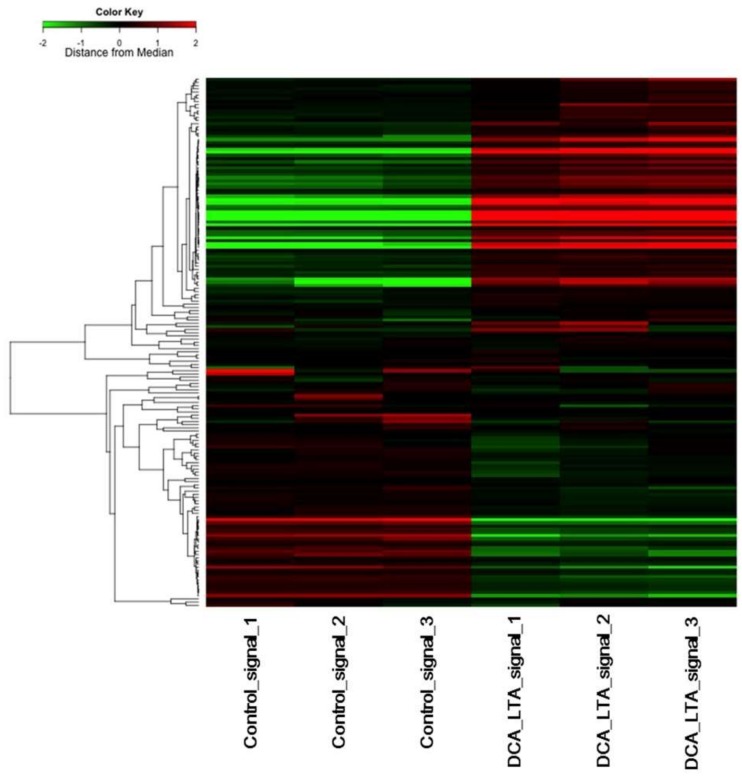
Clustering diagram of gene trees and heatmap of the tumor necrosis factor (TNF) signaling pathway generated using the MeV software. LX-2 cells were cultured in Dulbecco’s modified Eagle’s medium (DMEM) with or without 300 μM deoxycholic acid (DCA) and 25 μg/mL lipoteichoic acid (LTA) for 48 h in triplicate. We used the hierarchical clustering method to sort the genes (the distance metric was “Pearson correlation” and the linkage method was “average linkage clustering”). Rows represent the genes, and columns represent the samples. Colors indicate the distance from the median of each row. Red and green blocks represent genes whose expression levels were higher or lower than in the control, respectively.

The enrichment of specific pathway components among the functionally regulated gene groups was characterized using the Kyoto Encyclopedia of Genes and Genomes (KEGG) pathway database. After exposure to DCA and LTA for 48 h, the major genes with altered expression levels were those involved in focal adhesion, systemic lupus erythematosus, alcoholism, extracellular matrix–receptor interactions, arrhythmogenic right-ventricular cardiomyopathy, the phosphoinositide 3-kinase (PI3K)/protein kinase B (Akt) signaling pathway, viral carcinogenesis, hypertrophic cardiomyopathy, influenza A, and the tumor necrosis factor (TNF) signaling pathway (Table 2). Using these pathways, a clustering diagram of gene trees and a heatmap of the TNF signaling pathway were generated. The heatmap shows gene expression patterns for samples exposed to 300 μM DCA and 25 μg/mL LTA, and indicates genes whose expression levels were significantly different from that in the control untreated cells (Figure 2).

**Figure 3:**
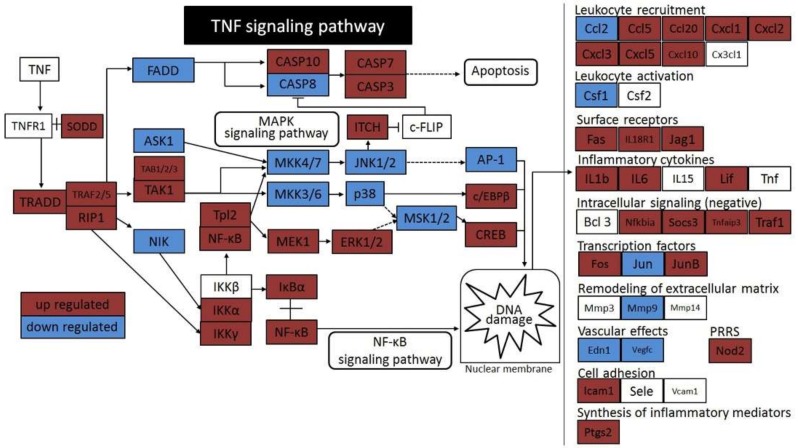
Schematic representation of deoxycholic acid (DCA) cytotoxicity mediated by the activation of the TNF signaling pathway. Solid arrows indicate direct interactions and broken arrows indicate indirect effects. Red represents statistically significant upregulation and blue represents statistically significant downregulation (*p* ≤ 0.05). Abbreviations: AP-1, activator protein-1; ASK1, apoptosis signal regulating kinase 1; Bcl-3, B-cell lymphoma 3; CASP, caspase; Ccl, C–C motif chemokine ligand; CEBPB, CCAAT/enhancer-binding protein beta; c-FLIP, cellular Fas-associated via death domain (FADD)-like interlukin (IL)-1β-converting enzyme-inhibitory protein; Csf, colony stimulating factor; Cx3cl1, C–X3–C motif chemokine ligand 1; Cxcl, C–X–C motif chemokine ligand; Edn1, endothelin 1; ERK, extracellular signal-related kinase; FADD, Fas associated via death domain; Fas, Fas cell surface death receptor; Fos, Fos proto-oncogene; ICAM1, intercellular adhesion molecule 1; IκB, inhibitor of nuclear factor kappa B (NF-κB); IKK, IκB kinase; ITCH, itchy E3 ubiquitin protein ligase; Jag1, Jagged1; JNK, c-Jun N-terminal kinase; Jun, Jun proto-oncogene; Lif, leukemia inhibitory factor; MAPK, mitogen-activated protein kinase; MEK1, MAPK/ERK kinase 1; MKK, mitogen-activated protein kinase kinase; MSK, mitogen- and stress-activated protein kinase; NF-κB, nuclear factor kappa B; NIK, NF-κB-inducing kinase; Nod2, nucleotide-binding oligomerization domain-containing 2; Ptgs2, prostaglandin-endoperoxide synthase 2; RIP1, receptor-interacting protein 1; Sele, selectin E; Socs3, suppressor of cytokine signaling 3; SODD, silencer of death domains; TAB, transforming growth factor beta (TGF-β)-activated kinase binding protein 1; TAK1, TGF-β-activating kinase 1; Tnfaip3, TNF-α-induced protein 3; TNFR1, TNF receptor superfamily member 1; Tpl2, tumor progression locus 2; TRADD, TNFR1-associated via death domain; TRAF, TNF receptor-associated factor; Vcam1, vascular cell adhesion molecule 1; Vegfc, vascular endothelial growth factor C.

Based on the KEGG pathway database, a schematic diagram (Figure 3) was obtained, which shows how the TNF signaling pathway might be affected by the treatment with DCA and LTA in this experiment. Within the TNF signaling pathway, many genes were affected significantly (*p* ≤ 0.05). The list of upregulated genes included those that encoded proteins such as TNF receptor 1 (TNFR1)-associated via death domain (TRADD), silencer of death domains (SODD), TNF receptor-associated factor (TRAF)2/5, receptor-interacting protein 1 (RIP1), transforming growth factor beta (TGF-β)-activated kinase binding protein (TAB)1/2/3, TGF-β-activating kinase (TAK)1, caspase (CASP)10, CASP7, CASP3, tumor progression locus 2 (Tpl2), nuclear factor kappa B (NF-κB), inhibitor of NF-κB (IκB) kinase (IKK)α/γ, mitogen-activated protein kinase (MAPK)/ extracellular signal-related kinase (ERK) kinase 1 (MEK1), IκBα, itchy E3 ubiquitin protein ligase (ITCH), ERK1/2, CCAAT/enhancer-binding protein beta (c/EBPβ), cyclic AMP (cAMP) response element-binding protein (CREB), C–C motif chemokine ligand (Ccl)5, Ccl20, C–X–C motif chemokine ligand (Cxcl)1, Cxcl2, Cxcl3, Cxcl5, Cxcl10, Fas cell surface death receptor (Fas), interleukin (IL)-18R1, Jagged1 (Jag1), IL-1b, IL-6, leukemia inhibitory factor (Lif), NF-κB inhibitor alpha (NF-κBiα), TNF-α-induced protein 3 (TNFAIP3), TRAF1, Fos proto-oncogene (Fos), Jun proto-oncogene (JUN)B, nucleotide-binding oligomerization domain-containing 2 (NOD2), intercellular adhesion molecule 1 (ICAM1), and prostaglandin-endoperoxide synthase 2 (Ptgs2). Downregulated genes (shown in blue) encoded Fas-associated via death domain (FADD), apoptosis signal regulating kinase 1 (ASK1), NF-κB-inducing kinase (NIK), CASP8, mitogen-activated protein kinase kinase (MKK)4/7, MKK3/6, c-Jun N-terminal kinase (JNK)1/2, p38, mitogen- and stress-activated protein kinase (MSK)1/2, activator protein-1 (AP-1), Ccl2, colony stimulating factor (Csf)1, Jun, matrix metalloproteinase (MMP)9, endothelin 1 (Edn1), and vascular endothelial growth factor C (VEGF-C) proteins.

### 2.2. qRT-PCR for Quantification of TNF, TNFR1, and TRADD mRNAs

**Figure 4:**
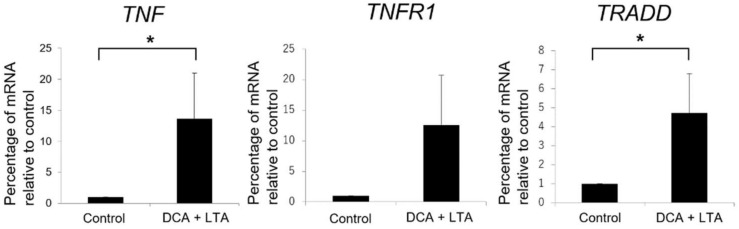
Effects of deoxycholic acid (DCA) and lipoteichoic acid (LTA) on *tumor necrosis factor* (*TNF*), *TNF receptor superfamily member 1* (*TNFR1*), and *TNFR1-associated via death domain* (*TRADD*) messenger RNA (mRNA) expression levels in LX-2 cells. The mRNA expression levels were normalized to those of β-actin and are presented as mean percentages relative to control ± standard deviation. The *p*-values were as follows: *TNF*, 0.036; *TNFR1*, 0.053; and *TRADD*, 0.048. *TNF* and *TRADD* mRNA expression levels were significantly higher in the DCA (300 µM) + LTA (25 μg/mL) group than in the control group treated with medium alone (* *p* ≤ 0.05).

Relative messenger RNA (mRNA) expression levels of *TNF* and *TRADD* in LX-2 cells were significantly higher in the group treated with DCA and LTA than in the control (DMEM) group (control, 1.0; *TNF*, 13.6 ± 7.4; *TRADD*, 4.7 ± 2.1) (Figure 4). *TNFR1* mRNA expression levels also appeared to increase (control, 1.0; *TNFR1*, 12.6 ± 8.1), but due to excessive variability, the nominal difference was not regarded as significant.

### 2.3. IL-6 Levels

**Figure 5:**
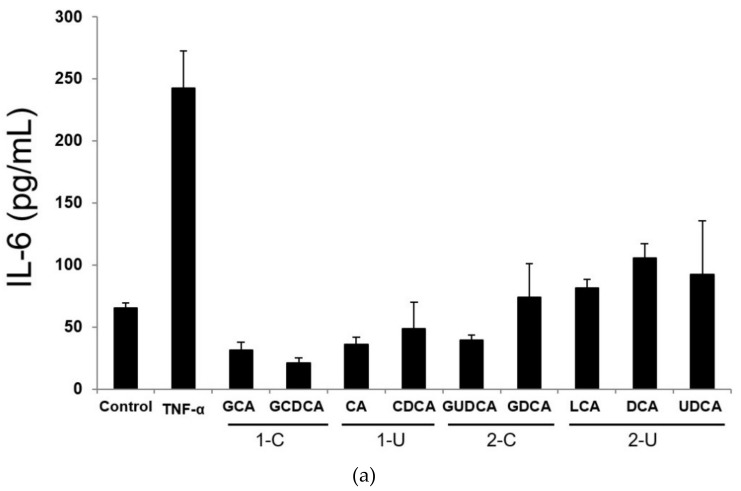

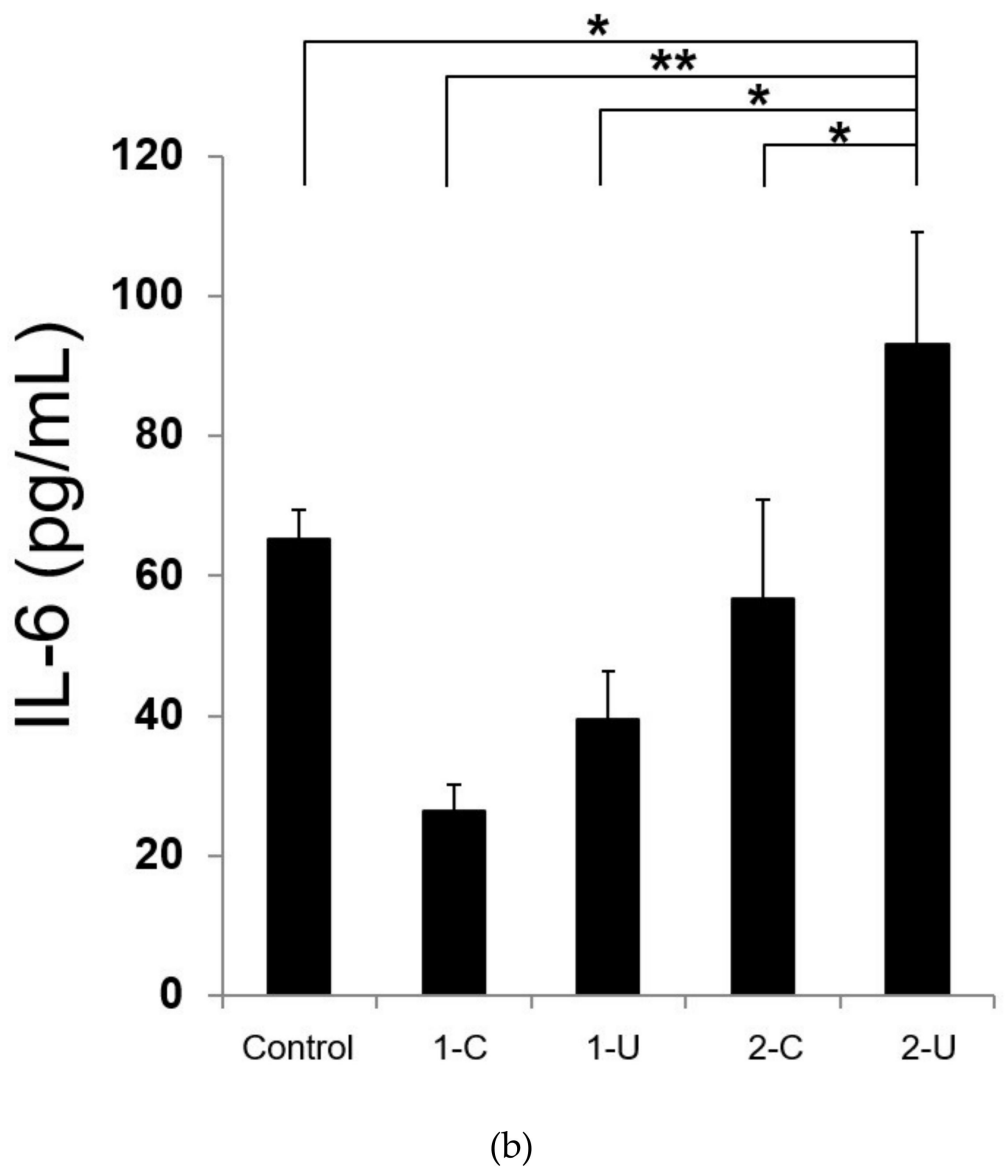
IL-6 levels in LX-2 cells. (**a**) IL-6 concentration in the supernatant of LX-2 cells. After treatment with TNF-α or nine bile acids (BAs), LX-2 cells were cultured for 48 h and the culture supernatant was collected. Supernatant levels of IL-6 were determined using an enzyme-linked immunosorbent assay. 1-C, primary conjugated BAs; 1-U, primary unconjugated BAs; 2-C, secondary conjugated BAs; 2-U, secondary unconjugated BAs. (**b**) IL-6 concentrations in the supernatant of LX-2 for BA classification. Data are expressed as the mean ± SD. Comparisons of different LX-2 cell treatments were carried out using one-way analysis of variance followed by Bonferroni’s correction (* *p* < 0.05; ** *p* < 0.01).

The following IL-6 concentrations (ng/mL) were observed in LX-2 cell supernatants after exposures to control medium, TNF-α, or BAs: control, 65.3 ± 4.0; TNF-α, 243 ± 29.8; glycolic acid (GCA), 31.6 ± 6.5; glycochenodeoxycholic acid (GCDCA), 21.3 ± 3.6; cholic acid (CA), 35.8 ± 6.2; chenodeoxycholic acid (CDCA), 48.9 ± 21.3; glycoursodeoxycholic acid (GUDCA), 39.4 ± 4.4; glycodeoxycholic acid (GDCA), 74.1 ± 27.1; lithocholic acid (LCA), 81.6 ± 7.1; DCA, 106 ± 11.6; and ursodeoxycholic acid (UDCA), 92.2 ± 43.6. IL-6 levels comprised, on average, 26.4 ± 3.8 pg/mL, 39.4 ± 6.9 pg/mL, 56.8 ± 14.2 pg/mL, and 93.0 ± 16.0 pg/mL in the supernatants of cells treated with primary conjugated BAs, primary unconjugated BAs, secondary conjugated BAs, and secondary unconjugated BAs, respectively (Figure 5a). Thus, IL-6 concentrations in the supernatants of cells treated with secondary unconjugated BAs were significantly higher than those in supernatants of cells from other groups (Figure 5b).

### 2.4. Immunofluorescent Staining

**Figure 6:**
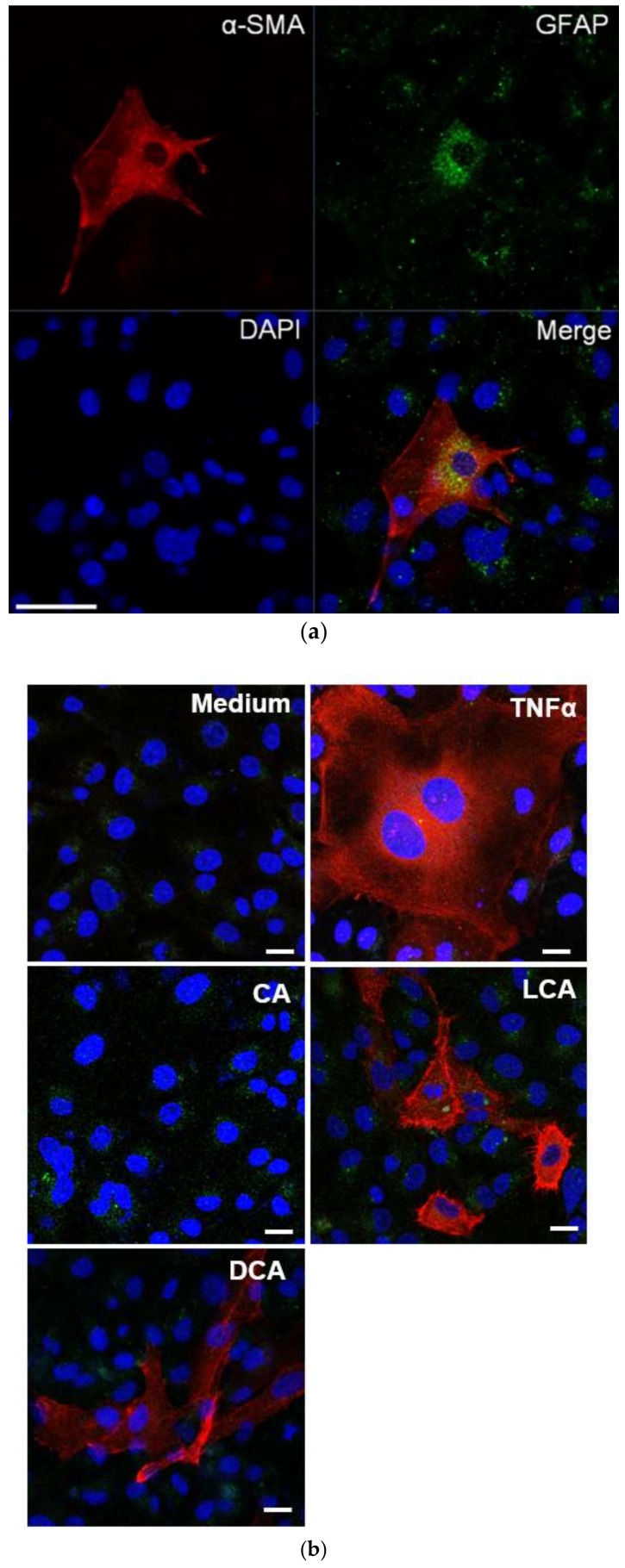
(**a**) Distribution of α-smooth muscle actin (α-SMA; red) and glial fibrillary acidic protein (GFAP; green) in LX-2 cells exposed to bile acids. LX-2 cells were cultured with 500 μM DCA and were immunostained for α-SMA and GFAP. Nuclei were stained with 4′,6-diamidino-2-phenylindole (DAPI; blue). Image overlays are shown. Scale bars, 50 µm. (**b**) Morphological changes in LX-2 cells exposed to different bile acids. LX-2 cells were treated with 500 µM cholic acid (CA), LCA, or DCA for 48 h. In LX-2 cells treated with control medium, few morphological changes were observed. In LX-2 cells treated with TNF-α, hypertrophy of the cytoplasm, a morphological change in which the subendothelial protrusion extended radially, was observed. In groups treated with primary conjugated BAs, primary unconjugated bile acids (Bas), and secondary conjugated BAs, almost no change in morphology was observed. In the group treated with secondary unconjugated BAs, cytoplasm enlargement was more prominent. Scale bars, 20 µm. For other BAs, see Supplementary Materials Figure S1.

Next, we examined expression levels of α-SMA and glial fibrillary acidic protein (GFAP) in LX-2 cells using immunofluorescence staining with antibodies and nuclear staining with 4′,6-diamidino-2-phenylindole (DAPI) (Figure 6a). GFAP was highly expressed in cells exposed to control medium, GCA, GCDCA, CA, CDCA, GUCDCA, and GDCA, indicating a quiescent state. The expression of α-SMA was low in those groups. However, α-SMA was highly expressed in the positive control group, where cells were exposed to 10 ng/mL TNF-α. In these cells, as well as in those treated with unconjugated secondary BAs (LCA, DCA, or UDCA), morphological changes such as hypertrophy of the cytoplasm and radial prolongation of subendothelial processes were observed (Figure 6b). The experiments were repeated in triplicate, and the above results were reproducible.

### 2.5. Flow Cytometry Analysis

**Figure 7:**
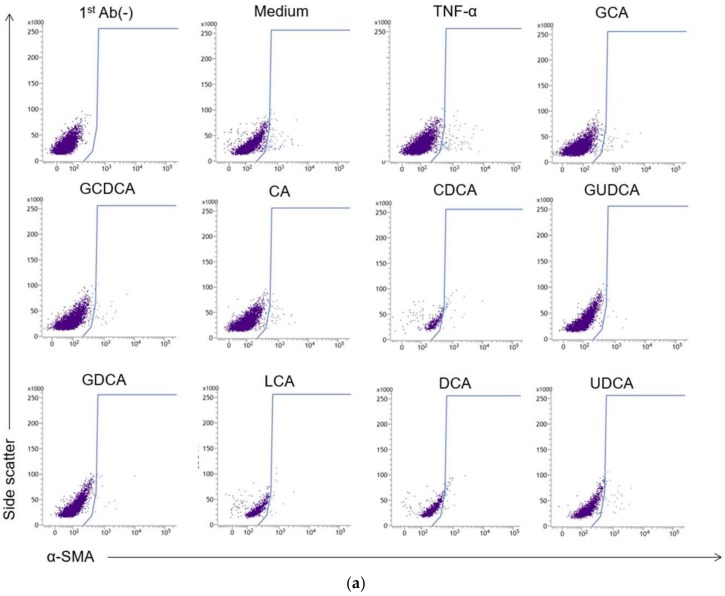

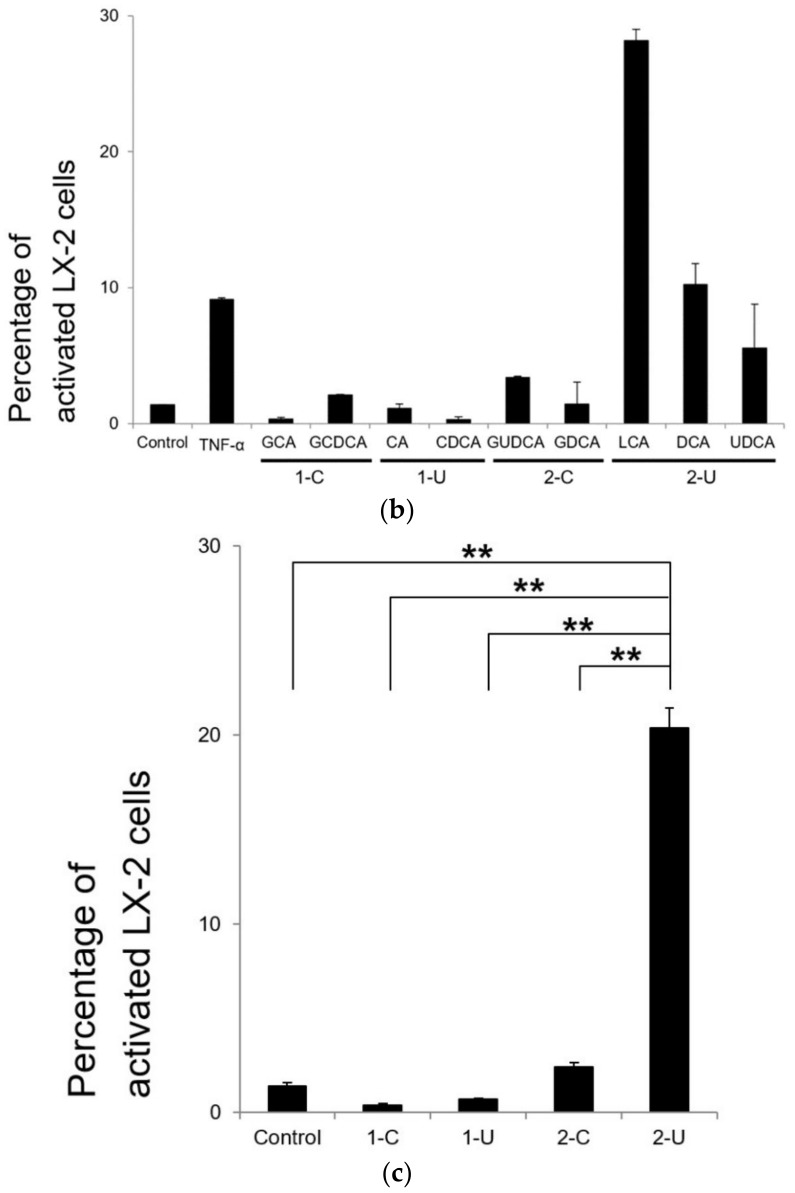
Activation of LX-2 cells. (**a**) Representative plots of α-smooth muscle actin (α-SMA) in LX-2 cells cultured with or without 10 ng/mL TNF-α and various BAs at a concentration of 500 µM for 48 h that were analyzed using flow cytometry. Gating was performed using the group without the primary antibody (1stAb(−)) as control. Hepatic stellate cell (HSC) population was analyzed by α-SMA (x-axis) and side scatter (y-axis). (**b**) Percentages of activated HSCs with high expression of α-SMA cultured with various bile acids were measured using flow cytometry. Data are expressed as the mean ± SD of 5–7 separate experiments. 1-C, primary conjugated BAs; 1-U, primary unconjugated BAs; 2-C, secondary conjugated BAs; 2-U, secondary unconjugated BAs. (**c**) Percentages of activated HSCs averaged per BA treatment group. Data are expressed as the mean ± SD. Comparisons of different LX-2 cell treatments were carried out using one-way analysis of variance followed by Bonferroni’s correction (** *p* < 0.01).

Fluorescence intensity and cell density of α-SMA were measured to evaluate the extent of activation of HSCs. Cells that were not exposed to the primary antibody were used as a control, and gating was performed to identify activated HSCs (Figure 7a). The percentages of activated HSCs are shown in Figure 7b,c. The percentage of cells with high α-SMA expression in the groups treated with secondary unconjugated BAs (LCA, DCA, UDCA) was significantly higher than that in cells exposed to other BAs (primary conjugated BAs, 0.38 ± 0.1; primary unconjugated BAs, 0.69 ± 0.2; secondary conjugated BAs, 2.4 ± 1.1; and secondary unconjugated BAs, 20.4 ± 2.6).

## 3. Discussion

Obesity not only elevates the risk of diabetes, dyslipidemia, hypertension, hyperuricemia, and myocardial infarction, but it also increases the incidence of cancer. Based on the results of epidemiological studies in Japan, obesity and overweight are almost certain to increase the risk of primary liver cancer [11,12]. Recently, liver cancer was shown to be promoted by BA metabolism of gut microbiota [13]. BAs produced from cholesterol absorb fatty acids and retinoids and excrete lipids and cholesterol. BAs are produced in the liver and are excreted into bile [14]. The excreted primary BAs (CA and CDCA) are unconjugated and deoxygenated by enterobacteria and are reabsorbed in the intestine as secondary BAs such as LCA, DCA, and UDCA [15]. BAs are further conjugated with glycine or taurine. The cycle of BAs from the intestine back to the liver is known as enterohepatic circulation [16]. In healthy individuals, only small quantities of BAs are found in peripheral circulation and urine. However, in obese or hepatobiliary patients, disturbances of synthesis and clearance by the liver and absorption by the intestine affect the level and the pattern of serum BAs [17].

Among the BAs, DCA was reported to be strongly associated with hepatocarcinogenesis [8], and LTA enhanced the toxicity of DCA [10]. Therefore, for the purpose of maximizing DCA toxicity, we performed DNA microarray analysis of cells exposed to a combination of DCA and LTA. Our data clearly showed upregulation of expression of many genes encoding TNF signaling pathway proteins. Furthermore, the majority of upregulated genes encoded components of the NF-κB signaling pathway. In addition, expression levels of many genes that encode SASP factors were also increased with high reproducibility by the treatment with DCA and LTA. These findings agree with previous reports about the ability of DCA to promote senescence of HSCs and expression of SASP factors [8,10].

Analysis of DNA microarray data from LX-2 cells treated with DCA and LTA suggested expression changes in many signaling pathways according to the KEGG pathway database (Table 2). We focused on the TNF signaling pathway and examined the influence of DCA on HSCs. DCA is classified as a secondary unconjugated BA (Table 1) that shows hydrophobic properties [18]. Hydrophobic Bas induce phosphorylation of reduced nicotinamide adenine dinucleotide phosphate (NADPH) oxidase and the formation of reactive oxygen species (ROS) [19]. It was suggested that conjugated BAs are preferred to unconjugated BAs as substrates of human organic-anion-transporting polypeptides in the liver [20]. Differential uptake of BAs may contribute to HSC activation response. In hepatocytes, the reactive oxygen species response results in epidermal growth factor receptor transactivation, and a similar mechanism may induce activation of HSCs [21,22,23].

In the pathway illustrated in Figure 3, MAPK signaling was not upregulated remarkably. In contrast, most genes that encoded NF-κB signaling pathway components were upregulated. In addition, qRT-PCR showed a significant increase in *TNF* and *TRADD* mRNA levels (Figure 4). This suggests that DCA acts on the NF-κB signaling pathway via TNF or TRADD, which are upstream elements of the TNF signaling pathway. Thus, the NF-κB signaling pathway might be significantly involved in the activation of HSCs, resulting in DNA damage.

Expression of TNF-α and infiltration of macrophages in adipose tissue are more pronounced in obese than in non-obese individuals, and expression changes are reduced with the improvement of obesity [24]. TNF-α is mainly produced by immune cells such as macrophages, including Kupffer cells in the liver, and is a type of inflammatory cytokine with cytotoxic and immunomodulatory activities [25]. TNF-α was also reported to have a carcinogenesis-promoting effect as serum TNF-α concentration is elevated in patients with advanced cancer and correlates inversely with prognosis after surgery [26]. After partial hepatectomy using a laparoscope [27], the NF-κB signaling pathway is activated, which is considered critical in the initiation of liver regeneration [28].

Intestine-derived lipopolysaccharide (LPS) triggers activation of a cytokine network starting from TNF-α. LPS binds to one of the pattern-recognition receptors, Toll-like receptor (TLR), which is involved in innate immunity. Among the nine subtypes of TLRs expressed in human HSCs [29], TLR-4 and TLR-2 are involved in defense against bacteria. TLR-4 forms a homodimer and recognizes LPS produced by Gram-negative bacteria, e.g., in cases of sepsis [30,31,32].

Recently, it was reported that TLR-2 is involved in HSC senescence and the expression of SASP factors [10]. TLR-2 forms heterodimers (TLR-2/1 or TLR-2/6) and recognizes lipoproteins and LTA of Gram-positive bacterial cell walls. TLR-2/1 directly recognizes pathogen-associated molecular patterns [33]. Furthermore, DCA and LTA cooperatively induce the expression of TLR-2 and SASP factors in senescent HSCs [10]. TLR-2 is activated by various ligands, including LTA, but interactions and relationships between DCA and TLR-2 are still unclear. Specifically, it is not known which heterodimer, TLR-2/1 or TLR-2/6, is preferentially involved or how they recognize DCA. The TLR-2 heterodimer recognizes mainly lipids and forms a hydrophobic channel in recognition of molecular patterns. Thus, it may tend to recognize or interact with hydrophobic secondary unconjugated BAs. Therefore, TLR-2 may play a vital role in HSC activation by DCA.

SASP-related genes (encoding Cxcl1, Cxcl2, Cxcl3, Cxcl10, Fas, IL-1b, and IL-6) were upregulated in this pathway. We measured the levels of IL-6 and found that they were significantly higher in the secondary unconjugated BA group than in the other BA groups. In the comparison between BAs, the maximum concentration to be added to clarify the difference was set at 500 µM. The reason for this is that when we used a concentration of more than 500 µM BA, the number of HSCs decreased drastically in our preliminary study. Activated HSCs showed a remarkable morphological transformation consistent with fibrosis (Figure 6a,b).

We also evaluated HSCs using immunofluorescence staining for α-SMA and flow cytometry analyses. In the secondary unconjugated BA groups, morphological changes, such as hypertrophy of cytoplasm and radial prolongation of subendothelial processes, were observed (Figure 6b). Flow cytometry results indicated that secondary unconjugated BAs, including DCA, caused fibrosis and potently activated HSCs.

Regarding the effect of the interaction of secondary unconjugated BAs on the activation of HSCs, two mechanisms are considered. The first is a direct detergent action on the cell membrane; because of the strong hydrophobicity of secondary unconjugated BAs, they have a high affinity for the cell membrane and high toxicity [34]. Secondly, the involvement of several receptors was indicated. The receptors that BAs act on were previously reported. For example, the cyclooxygenase-2 (COX2) pathway driven by the gut microbiota produced the lipid mediator, prostaglandin E2 (PGE2), in senescent HSCs in the tumor microenvironment, which suppressed antitumor immunity [10]. Farnesoid X receptor (FXR)-null mice rapidly developed liver tumors, indicating that FXR is a tumor suppressor [35,36]. Some groups reported that FXR was detected in HSCs [37]. Moreover, it was reported that BA signaling through FXR might influence differentiation of HSCs as the differentiation of stromal cells occurs in the bone marrow [38]. Recently, the crystal structure of the human *N*-acyl phosphatidylethanolamine-specific phospholipase D (NAPE-PLD) was reported and specific binding sites for DCA were discovered [39]. Regarding the degree of involvement of BAs in organs, excluding the liver, G protein-coupled receptor (TGR5) was reported as membrane-type BA receptor that is activated by BAs in the order of LCA > DCA > CDCA > CA in intestinal neuroendocrine cells, as well as the gall bladder, spleen, brown adipose tissue, macrophages, and cholangiocytes [40,41]. Therefore, BAs are pivotal signaling molecules stimulating FXR or TGR5 in many organs [15,42,43,44,45,46,47]. Additionally, HSCs activated in response to liver injury express TLR4, which promotes the activation of IκB kinase/NF-κB and JNK pathways in addition to the secretion of IL-6, transforming growth factor-β (TGF-β1), and monocyte chemoattractant protein 1 (MCP-1) [48]; however, it is unclear how these processes can be inhibited, which requires future studies.

TLR inhibitors that act upstream of TNF are under development for the treatment of sepsis, rheumatoid arthritis, systemic lupus erythematosus, and systemic sclerosis [49]. However, neither TLR-2 nor TLR-4 inhibitors were clinically commercialized because of the difficulty in controlling inflammatory cytokines such as IL-6 [50,51]. The chimeric monoclonal antibody, infliximab, which binds to TNF-α, is used clinically for treating rheumatoid arthritis and Crohn’s disease. However, hepatic impairment is a serious side effect of infliximab therapy. Thus, it is important to develop a drug that targets downstream molecules of the TNF signaling pathway.

Inhibiting the function of TNF-α with an anti-TNF-α treatment inhibits the activation of NF-κB [52,53]. Currently, the most effective approach to inhibit activation of the NF-κB signaling pathway involves strategies to selectively inhibit IKK activity [54]. However, clinical trials revealed toxicity problems, and no drug for this target was approved. Overall, the complexity of the NF-κB signaling pathway makes it difficult to target its components [55].

Another potential treatment target is the intestinal bacteria flora. Gram-positive bacteria, such as *Clostridium* cluster XI, contribute to an increase in the DCA level in obese mice. Thus, if the intestinal bacterial flora can be controlled effectively, it may be possible to suppress the activation of HSCs caused by DCA in the enterohepatic circulation. This indicates that the relationship between the BA fraction of stool and constituents of intestinal bacterial flora between normal individuals and patients with liver cirrhosis or hepatocellular carcinoma is important. This will require direct measurements of the concentrations of BAs in the portal vein (ongoing research).

In conclusion, secondary unconjugated BAs such as DCA induce the activation of human HSC. Appropriate control of the TNF signaling pathway and intestinal bacterial flora may help suppress inflammation of the liver and, ultimately, the occurrence of liver cancer.

## 4. Materials and Methods

### 4.1. Cell Culture

The human HSC line, LX-2, was purchased from Merck Millipore (Temecula, CA, USA). LX-2 cells were maintained in Dulbecco’s modified Eagle’s medium (DMEM; Wako Pure Chemical Industries, Osaka, Japan) containing 2% fetal bovine serum (FBS; Sigma-Aldrich, St. Louis, MO, USA), 1× penicillin/streptomycin (Merck Millipore), and 1× glutamine (Merck Millipore) in a humidified incubator in an atmosphere of 95% air and 5% CO_2_.

### 4.2. Reagents

All commercially available BAs and LTA were purchased from Sigma-Aldrich. These were glycolic acid, glycochenodeoxycholic acid, cholic acid, chenodeoxycholic acid, glycoursodeoxycholic acid, glycodeoxycholic acid, lithocholic acid, deoxycholic acid, and ursodeoxycholic acid (Table 1). All BAs were dissolved in dimethyl sulfoxide (DMSO)with final concentrations of the solvent less than 0.5%. TNF-α was purchased from Pepro Tech (Rocky Hill, NJ, USA).

### 4.3. DNA Microarray and Quantitative Real-Time Polymerase Chain Reaction (qRT-PCR)

LX-2 cells were seeded on a 24-well plate at 1 × 10^5^ cells/well in DMEM containing 2% FBS and cultured at 37 °C for 24 h. LX-2 cells were then cultured for 48 h in the medium with or without 300 μM DCA and 25 μg/mL LTA.

After exposure to DCA and LTA, total RNA was isolated from LX-2 cells using TRIzol Reagent (Cosmo Bio, Tokyo, Japan) and purified using the SV Total RNA Isolation System (Promega, Madison, WI, USA) according to the manufacturer’s instructions. RNA samples were quantified using an ND-1000 spectrophotometer (NanoDrop Technologies, Wilmington, DE, USA) and the quality was confirmed with a 2200 TapeStation (Agilent Technologies, Santa Clara, CA, USA).

RNA was amplified and labeled using a Low Input Quick Amp Labeling Kit, and hybridized using the G3 Human Gene Expression Microarray 8 × 60 K, v3, according to the manufacturer’s instructions (Agilent Technologies). All hybridized microarray slides were scanned by an Agilent scanner. Relative hybridization intensities and background hybridization values were calculated using Agilent Feature Extraction Software (9.5.1.1).

Raw signal intensities of all samples were log_2_-transformed and normalized by the quantile algorithm with “preprocessCore” library package [56] using the Bioconductor software [57]. We selected the probes, excluding control probes, where the detection *p*-values of all samples were less than 0.01, and used them to identify differentially expressed genes. Then, we applied the Linear Models for Microarray Analysis (Limma) package [58] of the Bioconductor software and obtained 58,201 genes. The criteria were that the Limma *p*-value was <0.05 and the absolute log-fold-change was >1 compared to LX-2 cells with or without DCA and LTA treatments. We identified 5390 genes in the same samples using these criteria.

### 4.4. qRT-PCR for mRNA Quantification of TNF, TNFR1, and TRADD

The qRT-PCR was performed as described previously [59] with a LightCycler system (Roche Diagnostics, Lewes, East Sussex, UK). The primers used were from Fasmac (Kanagawa, Japan): *TNF*, 5′-TCCTTCAGACACCCTCAACC-3′ (forward) and 5′-AGGCCCCAGTTTGAATTCTT-3′ (reverse); *TNFR1*, 5′-ACCAGGCCGTGATCTCTATG-3′ (forward) and 5′-CAGCTATGGCCTCTCACTCC-3′ (reverse); and *TRADD*, 5′-GCTTTGGAGATCAGCCTCAC-3′ (forward) and 5′-GTATCTGCAGCACCCAGGAT-3′ (reverse). Human β-actin (Fasmac) was amplified according to the manufacturer’s protocol as an internal control to allow for the quantitation of *TNF*, *TNFR1*, and *TRADD* amplification products. Data were analyzed using the LightCycler analysis software (Roche Diagnostics), and a standard curve correlating cycle number with the amount of products formed was plotted for each sequence of interest. The mRNA expression levels of *TNF*, *TNFR1*, and *TRADD* were then normalized to that of β-actin.

### 4.5. Enzyme-Linked Immunosorbent Assay

LX-2 cells were seeded on a 24-well plate at 1 × 10^5^ cells/well in DMEM containing 2% FBS and cultured at 37 °C for 24 h. After treatment with TNF-α or any of the nine BAs at the indicated concentrations, LX-2 cells were cultured for 48 h and the culture supernatant was collected. Supernatant levels of IL-6 were determined using an enzyme-linked immunosorbent assay (ELISA) according to the manufacturer’s instructions (Invitrogen). Absorption at 450 nm was read on a microplate reader (Bio-Rad Laboratories, Hercules, CA, USA).

### 4.6. Immunofluorescence Staining

LX-2 cells were seeded on a 24-well plate at 1 × 10^5^ cells/well in DMEM containing 2% FBS and cultured at 37 °C for 24 h. LX-2 cells were then cultured for an additional 48 h in the medium containing one of the nine BAs at a concentration of 500 μM. DMEM was used as negative control, and TNF-α (10 ng/mL) was used as positive control. LX-2 cells were fixed on the cover glass with 90% methanol. After blocking with normal rabbit serum (Vector Laboratories, Burlingame, CA, USA) and FBS on ice, LX-2 cells were reacted with a mouse monoclonal antibody against α-SMA (Biolegend, San Diego, CA, USA) and a rat monoclonal antibody against glial fibrillary acidic protein (GFAP; Invitrogen, Carlsbad, CA, USA) for 12 h. α-SMA is purportedly expressed in HSCs when they are activated, and GFAP is expressed when they are in the quiescent state. Anti-mouse immunoglobulin G (IgG)-Alexa 594 (Invitrogen) and anti-rat IgG-Alexa Fluor 488 (Invitrogen) were used as the secondary antibodies. Cells were mounted in VECTASHIELD^®^ Mounting Medium, the nucleus was stained with 4′,6-diamidino-2-phenylindole (DAPI; Vector Laboratories), and then the cells were sealed on a slide glass. Observations with a confocal laser microscope were carried out the next day. Images were obtained using a 40× oil objective lens (EC Plan-Neofluar 40×/1.30 Oil DIC; Carl Zeiss, Oberkochen, Germany). All cells were analyzed using an LSM710 confocal laser microscopy system and software that was built around the inverted microscope (Axio Observer Z1; Carl Zeiss).

### 4.7. Flow Cytometry Analysis

LX-2 cells were seeded on a six-well plate at a density of 5.0 × 10^5^ cells/well in DMEM containing 2% FBS and cultured at 37 °C for 24 h. After treatment with TNF-α or one of the nine BAs at the indicated concentrations, LX-2 cells were cultured at 37 °C for an additional 48 h. The cells were then fixed with 90% methanol, centrifuged at 250× *g* for 10 min, and the supernatant was removed. Blocking was performed with 2% rabbit serum, the cells were centrifuged again at 250× *g* for 10 min, and the supernatant was removed. LX-2 cells were then reacted with the anti-α-SMA antibody at 4 °C for 24 h and centrifuged at 250× *g* for 10 min. The supernatant was removed and reacted with anti-mouse IgG Alexa Fluor 488 for flow cytometry analysis. The cells were examined using a FACS Verse instrument (BD Biosciences, San Jose, CA, USA) and analyzed using the BD FlowJo™ software. The sensitivity of the fluorescence detectors was set daily using BD CS&T research beads (BD Biosciences). Forward- and side-scatter measurements were made using linear amplifiers, whereas fluorescence measurements were made with logarithmic amplifiers.

### 4.8. Statistical Analyses

All data are presented as the mean ± standard deviation and were analyzed using the SPSS II software (IBM, Chicago, IL, USA). The Student’s *t*-test for pairwise comparisons or one-way analysis of variance followed by Bonferroni’s post hoc test for multiple group comparisons was used. All statistical analyses were conducted with a significance level of α = 0.05 (*p* < 0.05).

## Figures and Tables

**Table 1:** Classification of bile acids (BAs) used in this study.

Primary conjugated BAs	Glycolic acid
	Glycochenodeoxycholic acid
Primary unconjugated BAs	Cholic acid
	Chenodeoxycholic acid
Secondary conjugated BAs	Glycoursodeoxycholic acid
	Glycodeoxycholic acid
Secondary unconjugated BAs	Lithocholic acid
	Deoxycholic acid
	Ursodeoxycholic acid

**Table 2:** Kyoto Encyclopedia of Genes and Genomes (KEGG) pathway functional classification of genes differentially expressed in LX-2 cells after treatment with deoxycholic acid (DCA) and lipoteichoic acid (LTA) for 48 h. Akt—protein kinase B; TNF—tumor necrosis factor; Rap1—Ras-proximate 1; NOD—nucleotide-binding oligomerization domain.

Term	Count	*p*-Value
Focal adhesion	78	1.5 × 10^−11^
Systemic lupus erythematosus	57	6.8 × 10^−11^
Alcoholism	67	5.1 × 10^−10^
Extracellular matrix–receptor interaction	38	6.9 × 10^−8^
Arrhythmogenic right ventricular cardiomyopathy	30	4.8 × 10^−6^
Phosphoinositide 3-kinase/Akt signaling pathway	95	7.3 × 10^−6^
Viral carcinogenesis	63	8.9 × 10^−6^
Hypertrophic cardiomyopathy	31	1.4 × 10^−5^
Influenza A	55	1.5 × 10^−5^
TNF signaling pathway	38	2.0 × 10^−5^
Rheumatoid arthritis	33	2.8 × 10^−5^
Rap1 signaling pathway	62	4.1 × 10^−5^
Cytokine–cytokine receptor interaction	66	5.9 × 10^−5^
NOD-like receptor signaling pathway	23	9.7 × 10^−5^
Protein digestion and absorption	31	1.9 × 10^−4^
Pathways in cancer	99	2.1 × 10^−4^
Legionellosis	22	2.2 × 10^−4^
Transcriptional misregulation in cancer	49	4.0 × 10^−4^
Proteoglycans in cancer	56	4.5 × 10^−4^
Dilated cardiomyopathy	29	4.7 × 10^−4^

The database for annotation, visualization, and integrated discovery (DAVID) version 6.8 functional annotation bioinformatics microarray analysis software was used to obtain KEGG pathway functional classifications. Only KEGG pathway terms for classifications that showed statistically significant differences in the expression of genes in cells treated with DCA and LTA compared to that in control cells are shown (*p* ≤ 0.0005).

## Supplementary Materials

Supplementary materials can be found at http://www.mdpi.com/1422-0067/19/10/3043/s1.

## Abbreviations

α-SMA	α-smooth muscle actin
BA	bile acid
CA	cholic acid
CDCA	chenodeoxycholic acid
DCA	deoxycholic acid
GCA	glycolic acid
GCDCA	glycochenodeoxycholic acid
GDCA	glycodeoxycholic acid
GUDCA	glycoursodeoxycholic acid
HSCs	hepatic stellate cells
KEGG	Kyoto Encyclopedia of Genes and Genomes
LCA	lithocholic acid
LPS	lipopolysaccharide
LTA	lipoteichoic acid
SASP	senescence-associated secretory phenotype
TLR	Toll-like receptor
TNF	tumor necrosis factor
UDCA	ursodeoxycholic acid
