# Supplementary material for: Secondary Unconjugated Bile Acids Induce Hepatic Stellate Cell Activation
Source: Int J Mol Sci. 2018 Oct 5;19(10):3043. doi: 10.3390/ijms19103043 (PMC6213941; doi:10.3390/ijms19103043)
Supplement: Supplementary file 1 [file ijms-19-03043-s001.pdf]

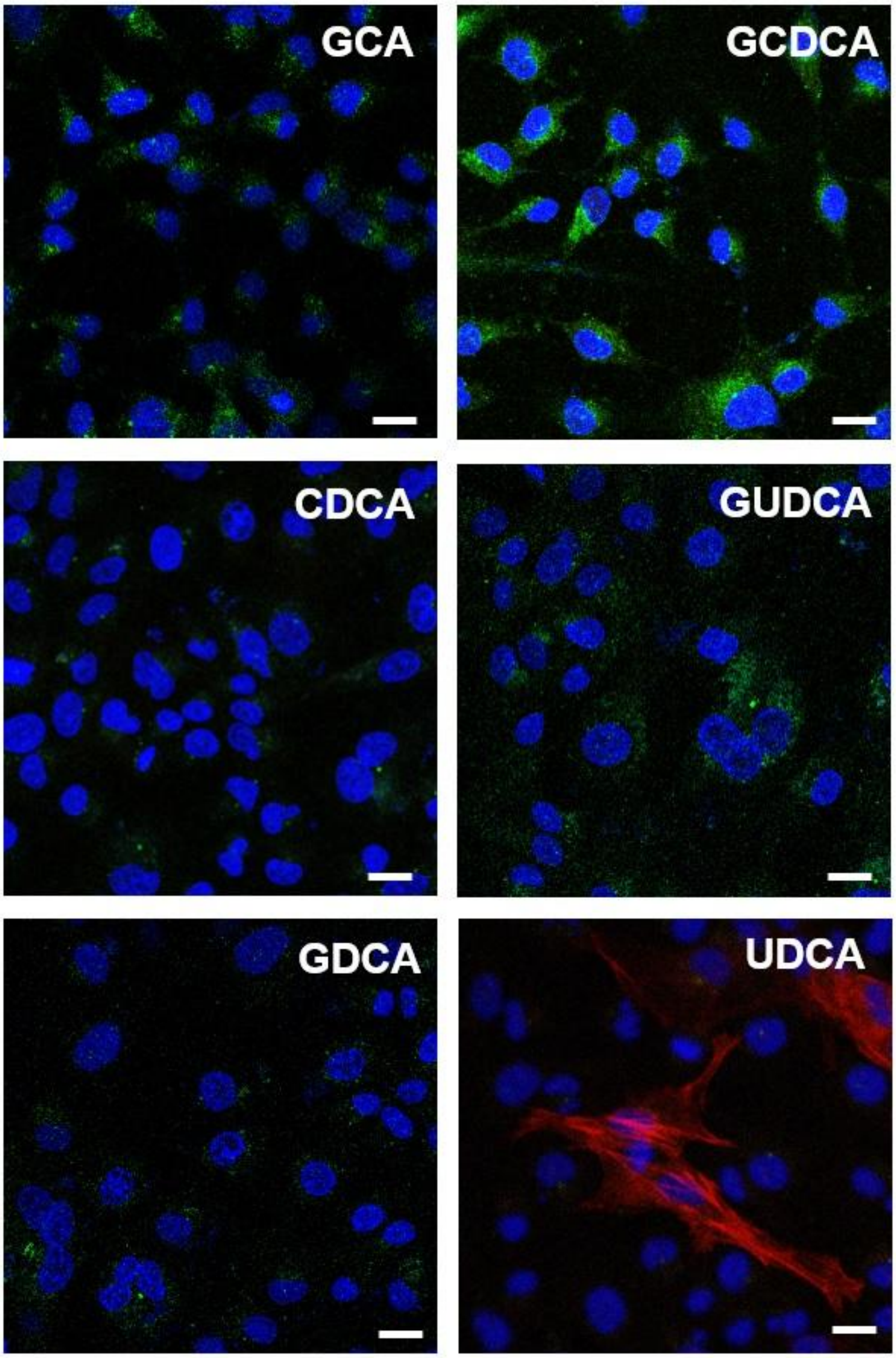

Figure S1. Morphological changes in LX-2 cells exposed to bile acids. LX-2 cells were treated with 500 μM glycolic acid (GCA), glycochenodeoxycholic acid (GCDCA), chenodeoxycholic acid (CDCA), glycoursodeoxycholic acid (GUDCA), glycodeoxycholic acid (GDCA), or ursodeoxycholic acid (UDCA) for 48 h. Scale bars, 20 μm.
